# Draft Genome Sequence of a *Clostridium botulinum* Isolate from Water Used for Cooling at a Plant Producing Low-Acid Canned Foods

**DOI:** 10.1128/genomeA.00200-12

**Published:** 2013-02-14

**Authors:** Uma Basavanna, Narjol Gonzalez-Escalona, Ruth Timme, Shomik Datta, Brianna Schoen, Eric W. Brown, Donald Zink, Shashi K. Sharma

**Affiliations:** a Division of Microbiology, Office of Regulatory Science; b Office of Center Director, Center for Food Safety and Applied Nutrition, Food and Drug Administration, College Park, Maryland, USA

## Abstract

*Clostridium botulinum* is a pathogen of concern for low-acid canned foods. Here we report draft genomes of a neurotoxin-producing *C. botulinum* strain isolated from water samples used for cooling low-acid canned foods at a canning facility. The genome sequence confirmed that this strain belonged to *C. botulinum* serotype B1, albeit with major differences, including thousands of unique single nucleotide polymorphisms (SNPs) compared to other genomes of the same serotype.

## GENOME ANNOUNCEMENT

*Clostridium botulinum* is a Gram-positive, spore-forming anaerobic bacterium that produces botulinum neurotoxin (BoNT) (1). Intoxication with the potent BoNT gives rise to the serious paralytic illness botulism in humans and is a serious concern for food safety. The neurotoxins produced by these organisms have been differentiated into seven serotypes, designated by the letters A through G (4). Four of the seven serotypes, A, B, E, and F, cause human botulism, with the vast majority of cases due to serotypes A and B (2). *C. botulinum* is the pathogen of concern for low-acid canned foods (LACF). An earlier investigative study from our lab revealed violations of LACF regulations as well as the finding of *C. botulinum* spores in the cooling water system at a canning facility (3). Two strains were isolated from water samples, INV-440848-Sub#4 (CFSAN001627) and INV-440848-Sub#16 (CFSAN001628). These strains produced two different toxins (A/B and A/F). To gain more information regarding these strains, their genomes were shotgun sequenced. Strain INV-440848-Sub#4 produced too many contigs and will be resequenced by use of other methodology that allows reducing the number of contigs (e.g., mate pair). Only the genome of INV-440848-Sub#16 produced discrete contigs, and this is the genome reported here.

The isolate was sequenced using Ion Torrent (PGM) sequencing with the 200-bp read chemistry (Life Technologies) according to manufacturer’s instructions at 30× coverage. Genomic DNA from each strain was isolated from overnight cultures with DNeasy blood and tissue kit (Qiagen, Valencia, CA). We constructed the libraries using 2 µg of genomic DNA, previously sheared using a Covaris (Covaris, Inc., Woburn, MA) following a protocol to generate an average of 200 bp, and the SPRI works Library Preparation System III (Beckman Coulter, Indianapolis, IN). The resultant libraries were diluted to the recommended concentrations, determined by quantitative PCR (qPCR) (Life Technologies) according to the manufacturer’s instructions, to be used as templates for emulsion PCR (emPCR). The resultant enriched emPCR was loaded onto a 318 chip and sequenced using an Ion PGM 200 sequencing kit according to the manufacturer’s instructions. The identification of this strain was performed using a reference mapping approach using the CLC Genomics Workbench software version 5.5.1 (CLC bio, Germantown, MD) against all of the *C. botulinum* genomes available in the NCBI database. The strain was identified as *C. botulinum* type B1. The genomic sequence contigs for strain INV-440848-Sub#16 were *de novo* assembled using the CLC Genomics Workbench. The G+C mol% of this strain was 28.1%, which is similar to the reported GC content for other *C. botulinum* strains. INV-440848-Sub#16 has 246 contigs, ranging from 550 to 199,101 bp. This strain contained a plasmid highly similar to the one in *C. botulinum* B1 Okra. The contigs were reorganized using the *C. botulinum* B1 strain Okra for INV-440848-Sub#16 using Mauve (http://gel.ahabs.wisc.edu/mauve). Sequences were annotated using the NCBI Prokaryotic Genomes Automatic Annotation Pipeline (PGAAP, http://www.ncbi.nlm.nih.gov/genomes/static/Pipeline.html).

A detailed report of the phylogenetic analysis of the draft genomes will be included in a future publication.

### Nucleotide sequence accession number.

The draft genome sequence of the *C. botulinum* strain is available in GenBank under accession number AMXJ00000000 (CFSAN001628).
